# Supplementary material for: Mitochondrial ATP fuels ABC transporter-mediated drug efflux in cancer chemoresistance
Source: Nat Commun. 2021 May 14;12:2804. doi: 10.1038/s41467-021-23071-6 (PMC8121950; doi:10.1038/s41467-021-23071-6)
Supplement: Supplementary file 1 — Supplementary Information [file 41467_2021_23071_MOESM1_ESM.pdf]

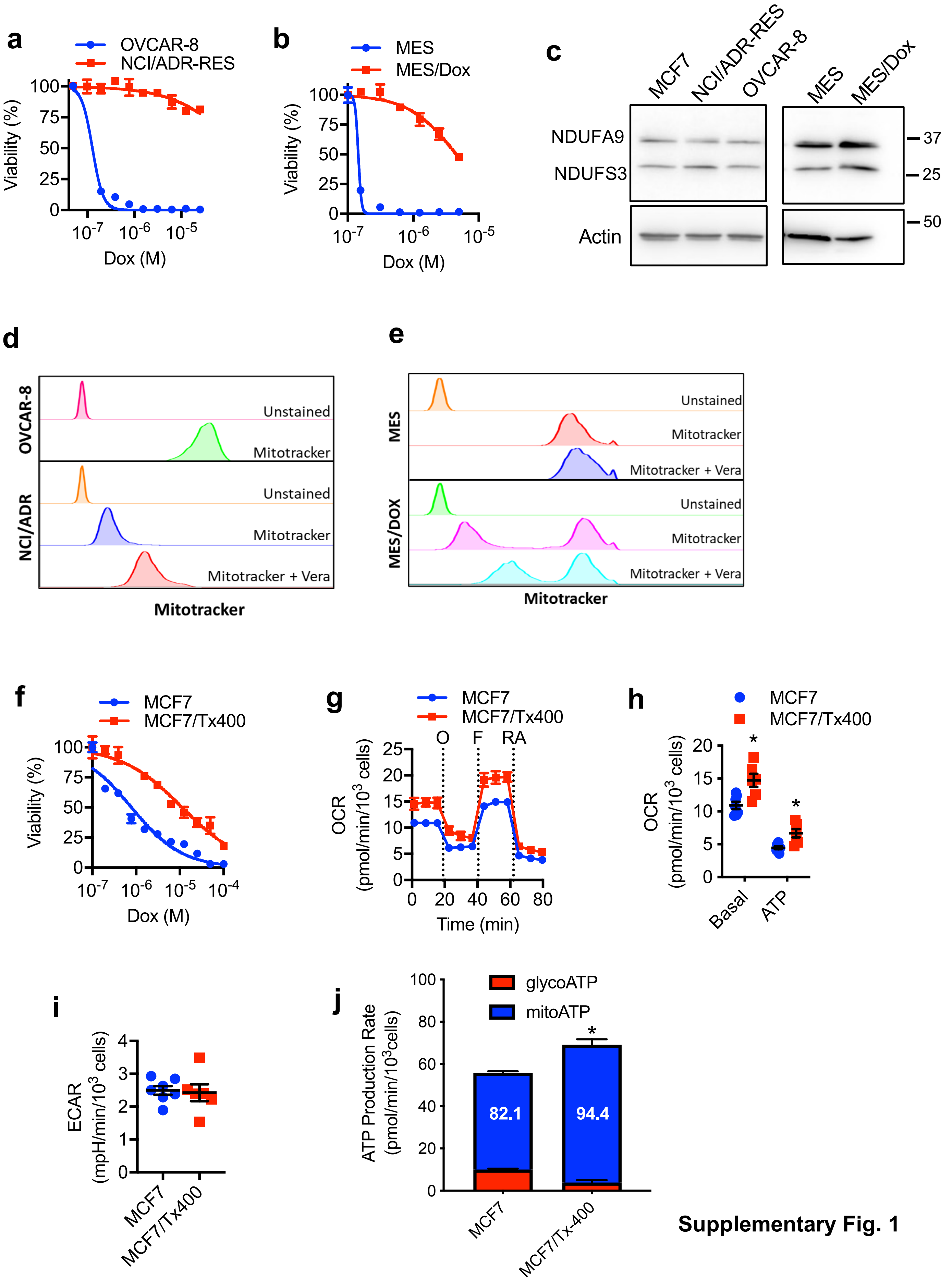

Supplementary Fig. 1

Supplementary Figure 1. Mitochondrial respiration in chemosensitive and chemoresistant cancer cells. (**a)** Viability of OVCAR-8 (n=4) and NCI/ADR-RES (n=4) cells in the presence of increasing concentrations of doxorubicin (Dox) as determined by MTT assay. (**b**) Viability of MES (n=6) and MES/Dox (n=6) cells in the presence of increasing concentrations of doxorubicin as determined by MTT assay. (**c**) Western blot analysis for the Complex I subunits NDUFA9 (about 36 kDa) and NDUFS3 (about 26 kDa) in MCF7 (chemosensitive), NCI/ADR-RES (chemoresistant), OVCAR-8 (chemosensitive), MES (chemosensitive), and MES/Dox (chemoresistant) cell lines. Numbers indicate the molecular markers (KDa). (**d and e**) Mitochondrial mass as determined by MitoTracker staining in NCI/ADR-RES and OVCAR-8 (d) or MES and MES/Dox (e) cell lines by flow cytometry analysis. Since Mitotracker could be used and effluxed by ABC transporters in chemoresistant cells, staining was performed in the absence or presence of Verapamil (10 mM), a broad inhibitor of ABC transporters. (**f)** Viability of MCF7 (n=4) and MCF7/Tx400 (n=4) cells in the presence of increasing concentrations of doxorubicin as determined by MTT assay. (**g** and **h**) OCR of MCF7 and MCF7/Tx400 cells were determined as described using the MitoStress assay (Seahorse/Agilent). (**h**) Basal and ATP-linked values are shown. MCF7 (n=7) and MCF7/Tx400 (n=6) cells for all. p=0.005, 0.004 by unpaired *t* test. (**i**) Baseline ECAR of MCF7 (n=7) and MCF7/Tx400 cells (n=6) as determined by extracellular flux analysis. p=0.8022 by unpaired *t* test (**j)** Mitochondrial and glycolytic ATP production rates in MCF7 and MCF7/Tx400 cells as determined by Seahorse ATP Production Rate Test. p=0.0001 by two-way ANOVA. Mean ± SEM is provided for all figure (n ≥ 4). * denotes p<0.05.

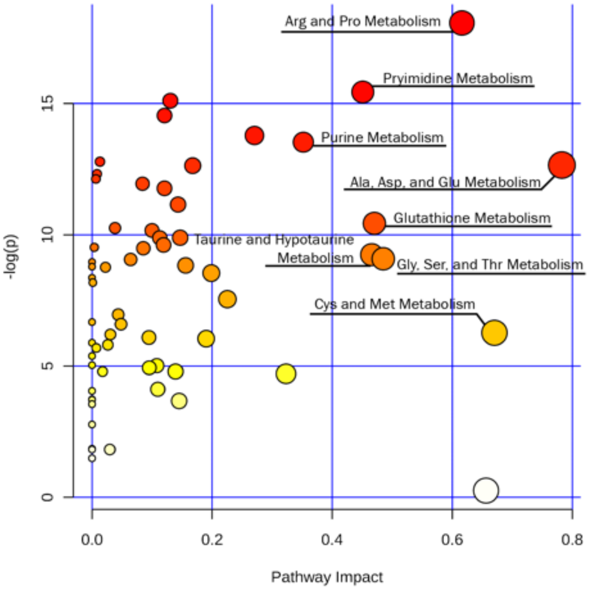

Supplementary Figure 2. Metabolic profile in chemoresistant cancer cells and summary plot of Metabolite Sets Enrichment Analysis (MSEA). Altered metabolic pathways in NCI/ADR-RES cells relative to OVCAR-8 cells, based on the statistically significant metabolites (unpaired t-test analysis). The p values for the metabolic pathways are color coded, white representing the least significant, and red the highly significant. Pathway impact represents the number of metabolites that support the assignment of a pathway, with higher number of metabolites associated with higher pathway impact.

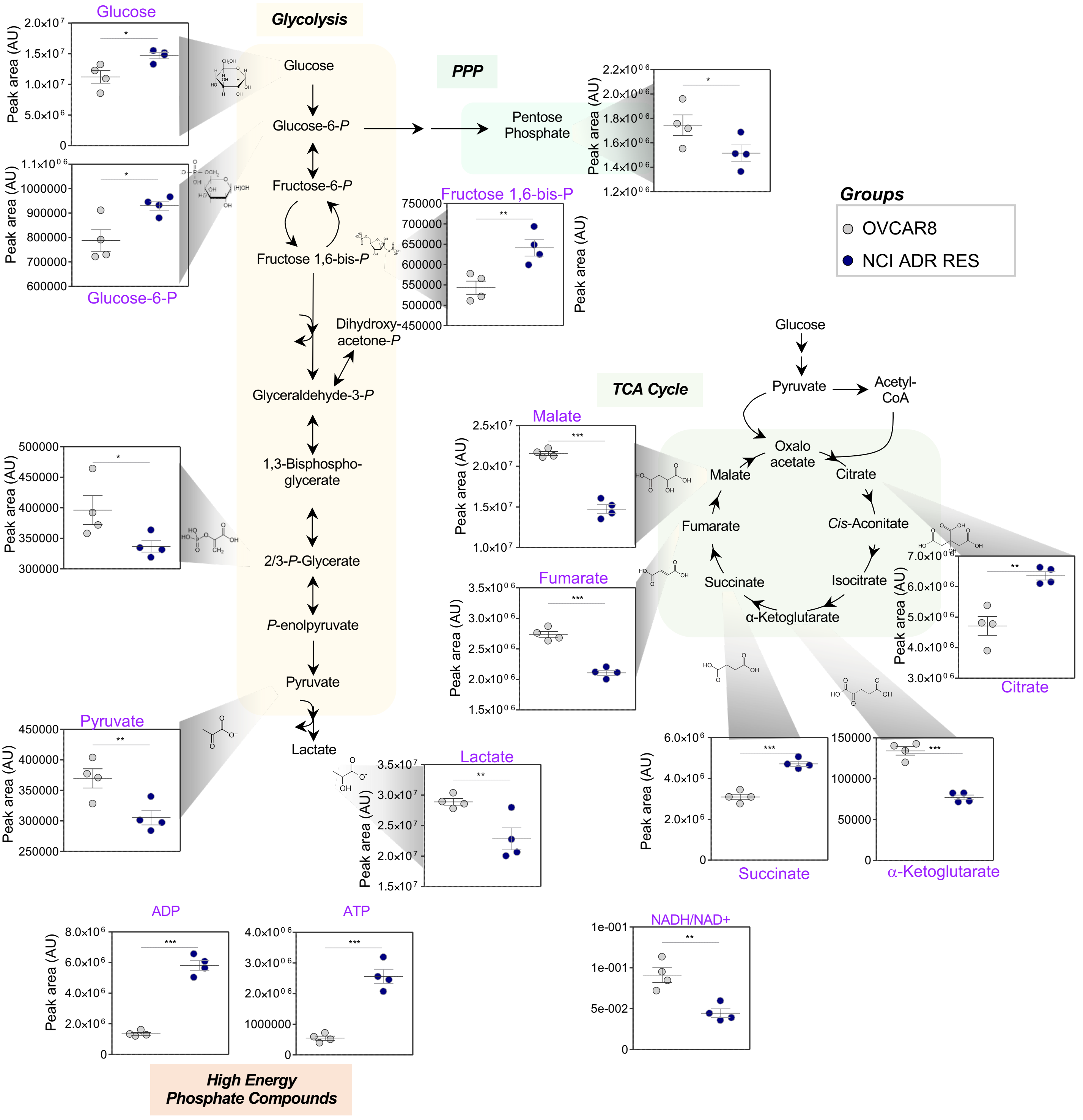

Supplementary Fig. 3. Peak area values (AU, arbitrary units) for metabolic intermediates in OVCAR-8 (n=4) and NCI/ADR-RES cells (n=4) as determined by mass spectrometry based metabolomics, corresponding to the heatmap shown in Fig. 1i. Mean ± SD provided. * denotes $p< 0.05$ by unpaired one-sided *t* test.

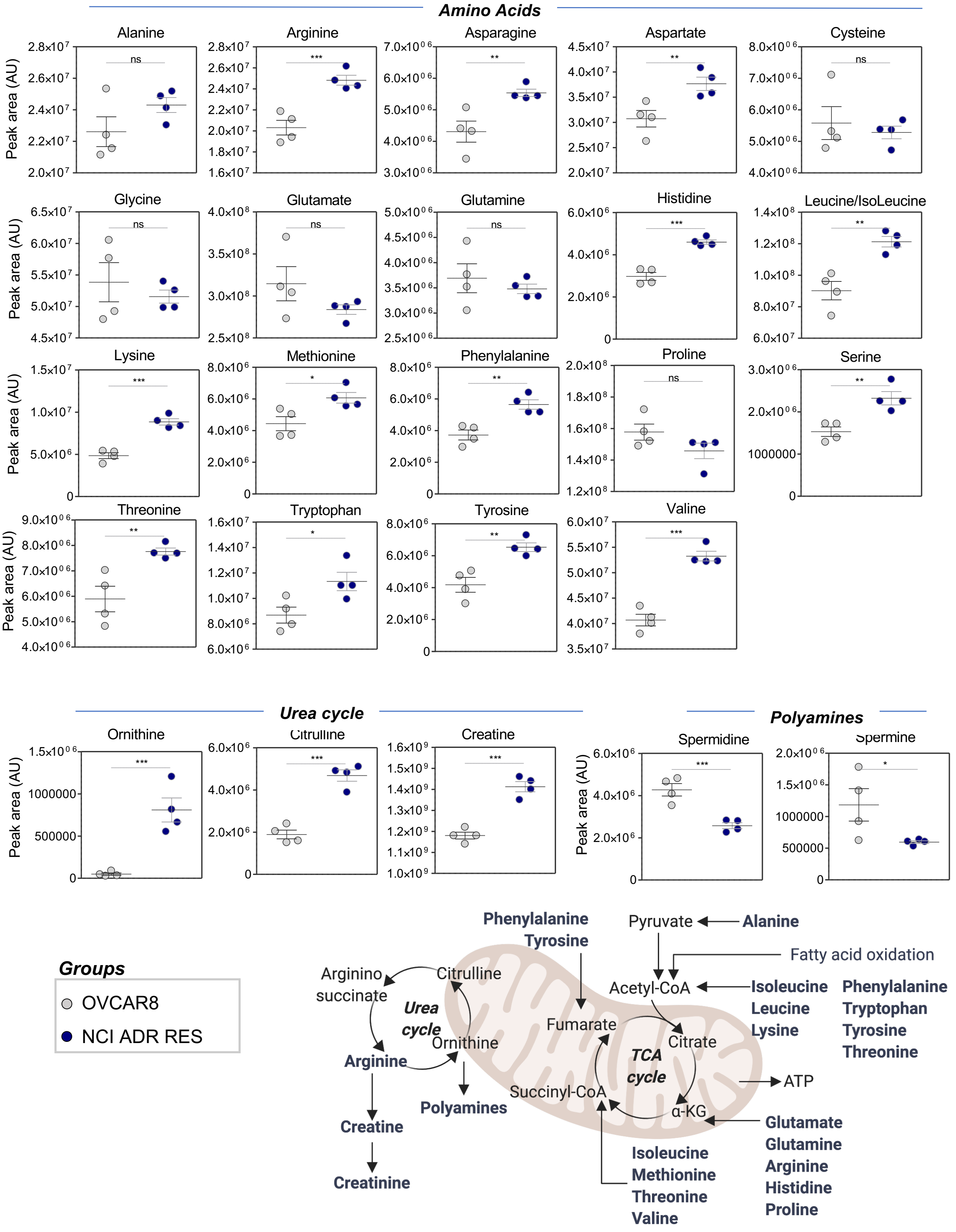

Supplementary Fig. 4. Peak area values (AU, arbitrary units). for amino acids and other metabolic intermediates in OVCAR-8 (n=4) and NCI/ADR-RES (n=4) cells as determined by mass spectrometry based metabolomics, corresponding to the heatmap shown in Fig. 1i. Mean ± SD provided. * denotes $p < 0.05$ by unpaired one-sided *t* test.

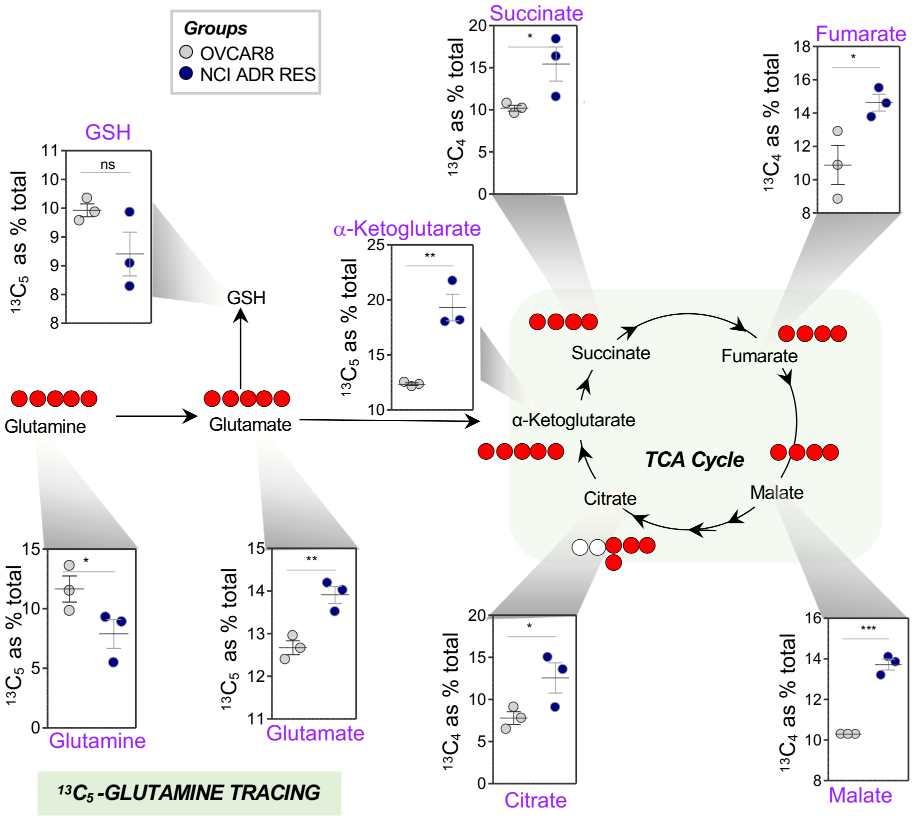

Supplementary Fig. 5. ^{13}C-$_5$Glutamine tracing analysis in OVCAR-8 (n=3) and NCI/ADR-RES (n=3) cells, after 24 h of incubation with labeled glutamine (650 μM). Y axes indicate the % of ^{13}C labeled isotopologues as a fraction of the total levels of each metabolite. Mean +/- SD is shown * denotes p <0.05; ** p<0.01; *** p<0.001 as determined by unpaired one-sided t-test analysis.

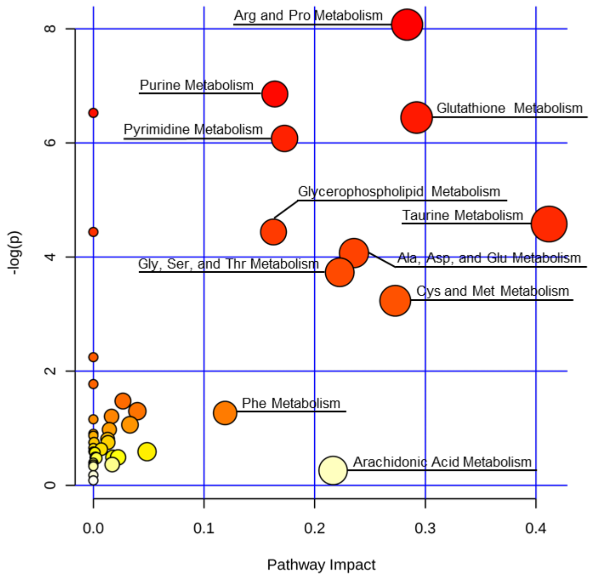

Supplementary Figure 6. **Metabolite Sets Enrichment Analysis (MSEA) in MES and MES/Dox.** Altered metabolic pathways in MES cells and MES/Dox cells, based on the statistically significant metabolites (unpaired t-test analysis). The p values for the metabolic pathways are color coded, white representing the least significant, and red the highly significant. Pathway impact represents the number of metabolites that support the assignment of a pathway, with higher number of metabolites associated with higher pathway impact.

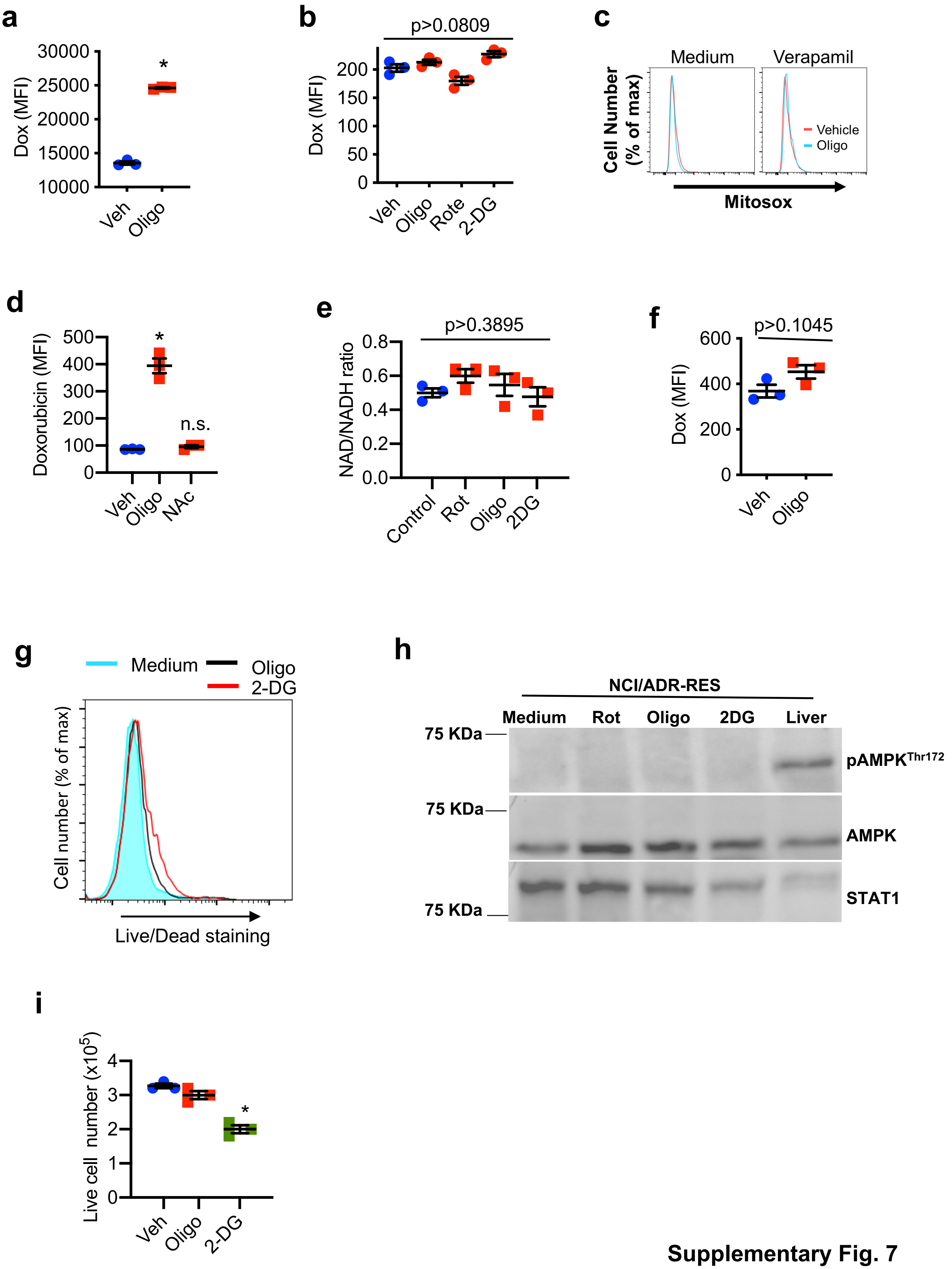

Supplementary Fig. 7

Supplementary Figure 7. Effect of NAC and metabolic inhibitors on accumulation of doxorubicin in cancer cells. (a) NCI/ADR-RES cells (n=8) were treated with or without (Veh) oligomycin (Oligo, 5 μM), followed by incubation with doxorubicin (Dox, 3 μM) for 30 min. Doxorubicin accumulation was examined by flow cytometry. Median fluorescence intensity (MFI). p=0.0001 by unpaired *t* test. **b**) OVCAR-8 cells (n=3) were treated with or without (Veh) oligomycin (Oligo, 5 μM), rotenone (Rote, 50 μM), or 2-deoxyglucose (2-DG, 50 mM) for 2 h followed by incubation with doxorubicin (Dox, 3 μM) for 3 h and doxorubicin accumulation was examined by flow cytometry. p=0.6765, 0.1016, 0.0809 by one-way ANOVA and Tukey's multiple comparisons test. (**c**) NCI/ADR-RES cells were treated with or without (Veh) oligomycin (Oligo, 5 μM) in the presence or absence of verapamil (Ver, 10 μM), inhibitor of ABC transporters. After 5 h, cells were stained with MitoSox and examined by flow cytometry. (**d**) NCI/ADR-RES cells (n=3) were treated with or without (Veh) oligomycin (Oligo, 5 μM), N-acetylcysteine (NAC, 5 mM) for 2 h followed by incubation with doxorubicin (Dox, 3 μM) for 3 h. p=0.0001, 0.9056 by one-way ANOVA and Tukey's multiple comparisons test. (**e**) NCI/ADR-RES cells (n=3) were treated with or without (Veh) oligomycin (Oligo, 5 μM), rotenone (Rote, 50 μM), or 2-deoxyglucose (2-DG, 50 mM) for 5 h and NAD/NADH ratio was determined following the manufacturer recommendation (BioVision). p=0.3895, 0.8405, 0.9733 by one-way ANOVA and Tukey's multiple comparisons test. (**f**) MES cells (n=3) were treated with or without (Veh) oligomycin (Oligo, 5 μM) for 2 h followed by incubation with doxorubicin (Dox, 3 μM) for 3 h. p=0.1045 by unpaired *t* test. (**g**) NCI/ADR-RES cells were treated with medium, oligomycin (Oligo, 5 μM), or 2-deoxyglucose (2-DG, 50 mM) for 5 h Cell death analysis using the Live/Death staining and flow cytometry. (**h**) Western blot analysis for phospho-AMPK, total AMPK (63 kDa) and STAT1 (as control, 84 kDa) in NCI/ADR-RES cells treated with rotenone, oligomycin or 2-DG for 5 h as described in (b). Representative of one independent experiment with two replicates. (**i**) NCI/ADR-RES cells (n=3) were treated with medium, oligomycin (Oligo, 5 μM), or 2-deoxyglucose (2-DG, 50 mM) for 18 h Live cell numbers were determined by Trypan Blue staining. p=0.2324, 0.0003 by one-way ANOVA and Tukey's multiple comparisons test. Mean +/- SEM is shown for all figures. *, denotes p<0.05 as determined by *t* test or one-way ANOVA and Tukey's multiple comparisons test.

Supplementary Fig. 8

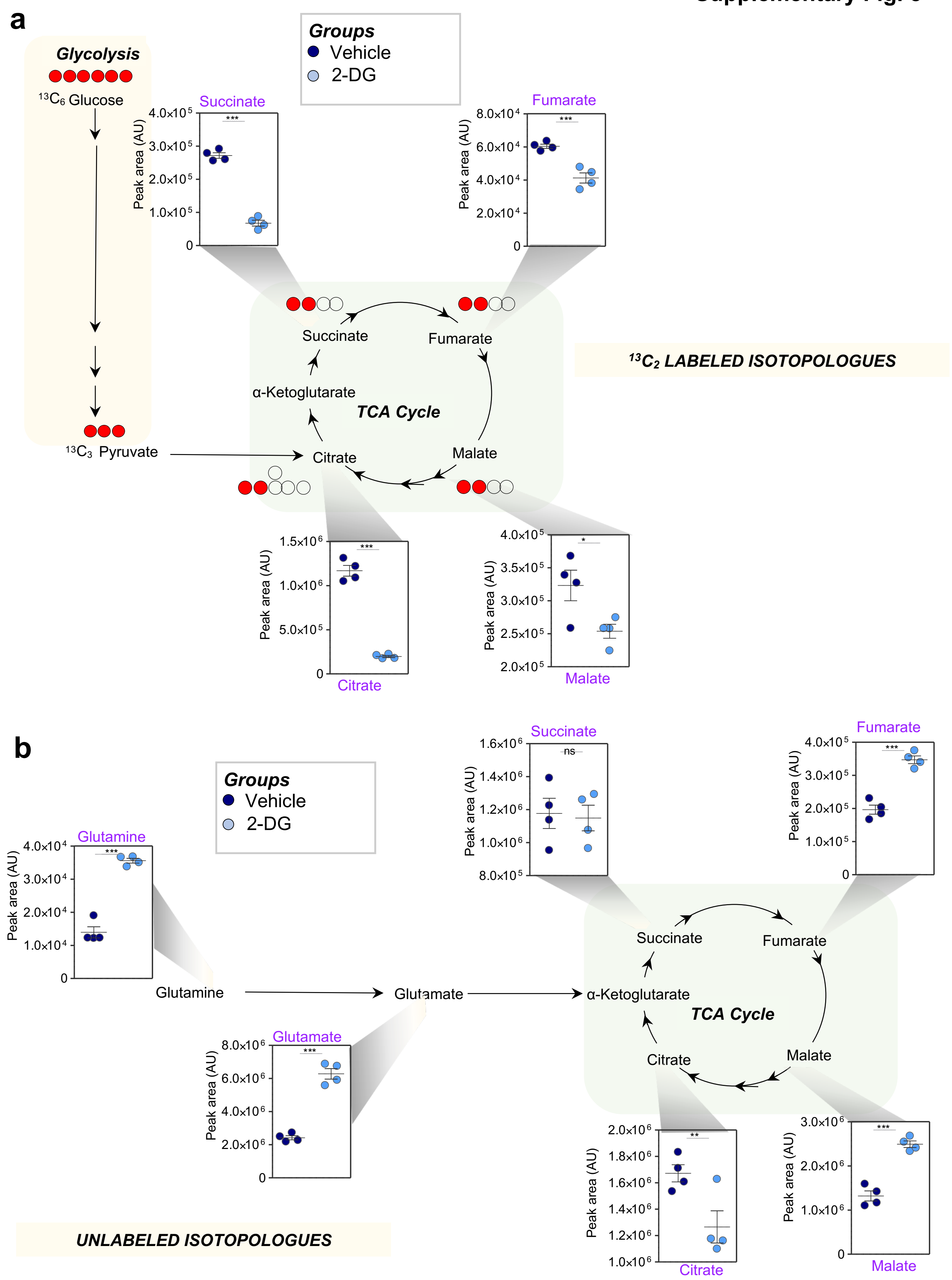

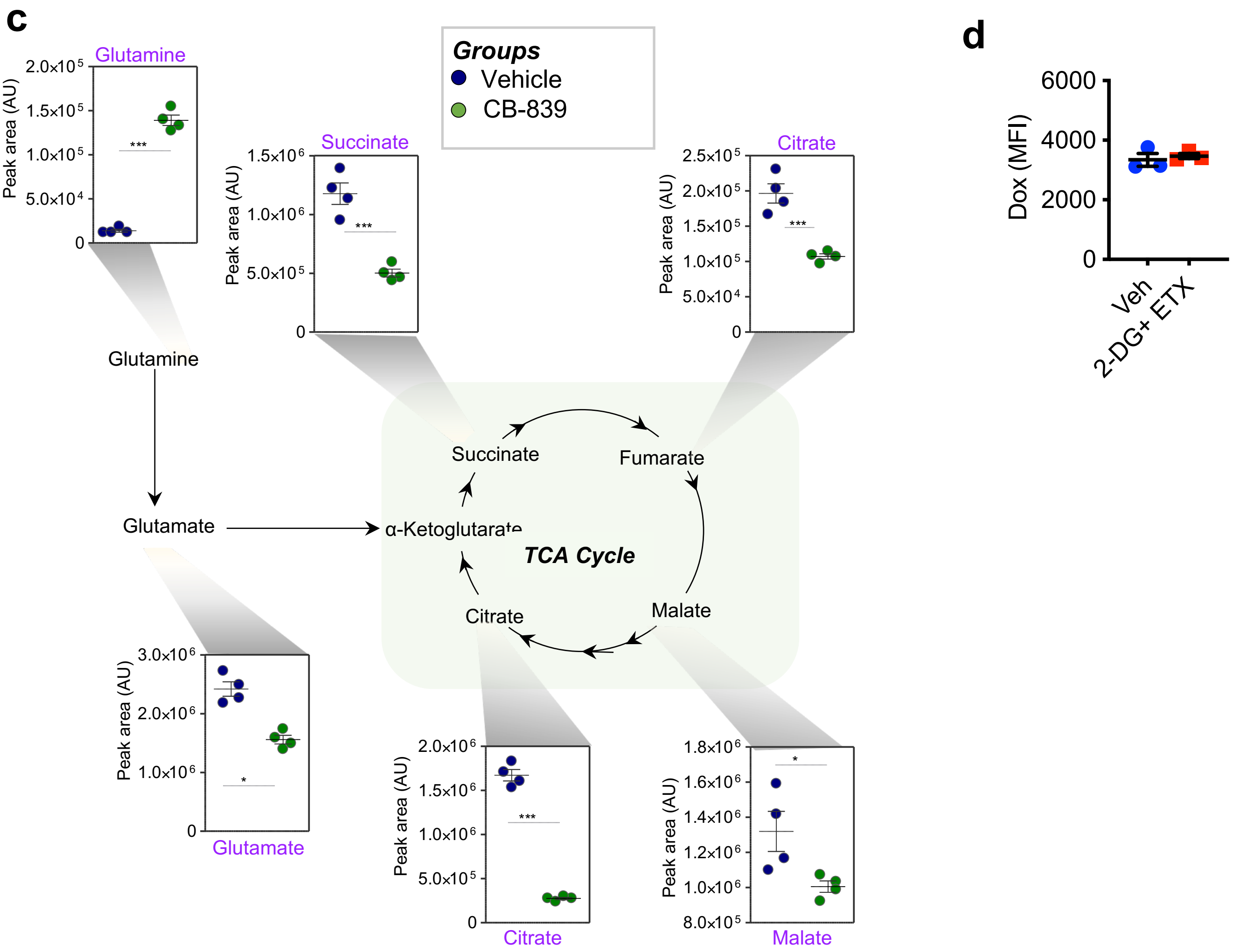

Supplementary Fig. 8. (a) and (b) NCI/ADR-RES cells (n=4) were incubated with $^{13}C_6$-Glucose (5.5 mM) in the absence (vehicle) or presence of 2-DG (50 mM). After 5 h, cells were harvested. ^{13}C-labeled isotopologues (only M+2 detected in this study) (**a**) and unlabeled isotopologues of Tricarboxylic acid (TCA) cycle metabolites (**b**) were determined by mass spectrometry-based metabolomics. Y axes indicate peak area values (arbitrary unis – AU). **(c)** NCI/ADR-RES cells (n=4) were treated with CB-839 (5 μM) or vehicle. After 5 h, cells were harvested and metabolites and the presence of the specified metabolites was determined by mass spectrometry based metabolomics. Peak area values (arbitrary units – AU) for the specified metabolites are shown. Mean ± SD is shown for **a, b** and **c**. * denotes $p < 0.05$ by unpaired one-sided *t* test. **(d)** NCI/ADR-RES cells (n=3) were incubated with 2-DG (50 mM) and etomoxir (ETX, 10 μg/ml) or vehicle for 2 h followed by incubation with doxorubicin (3 μM). Cells were then fixed and analyzed for doxorubicin fluorescence by flow cytometry. p=0.6066 by unpaired one-sided *t* test. Mean ± SEM is shown for (d) .

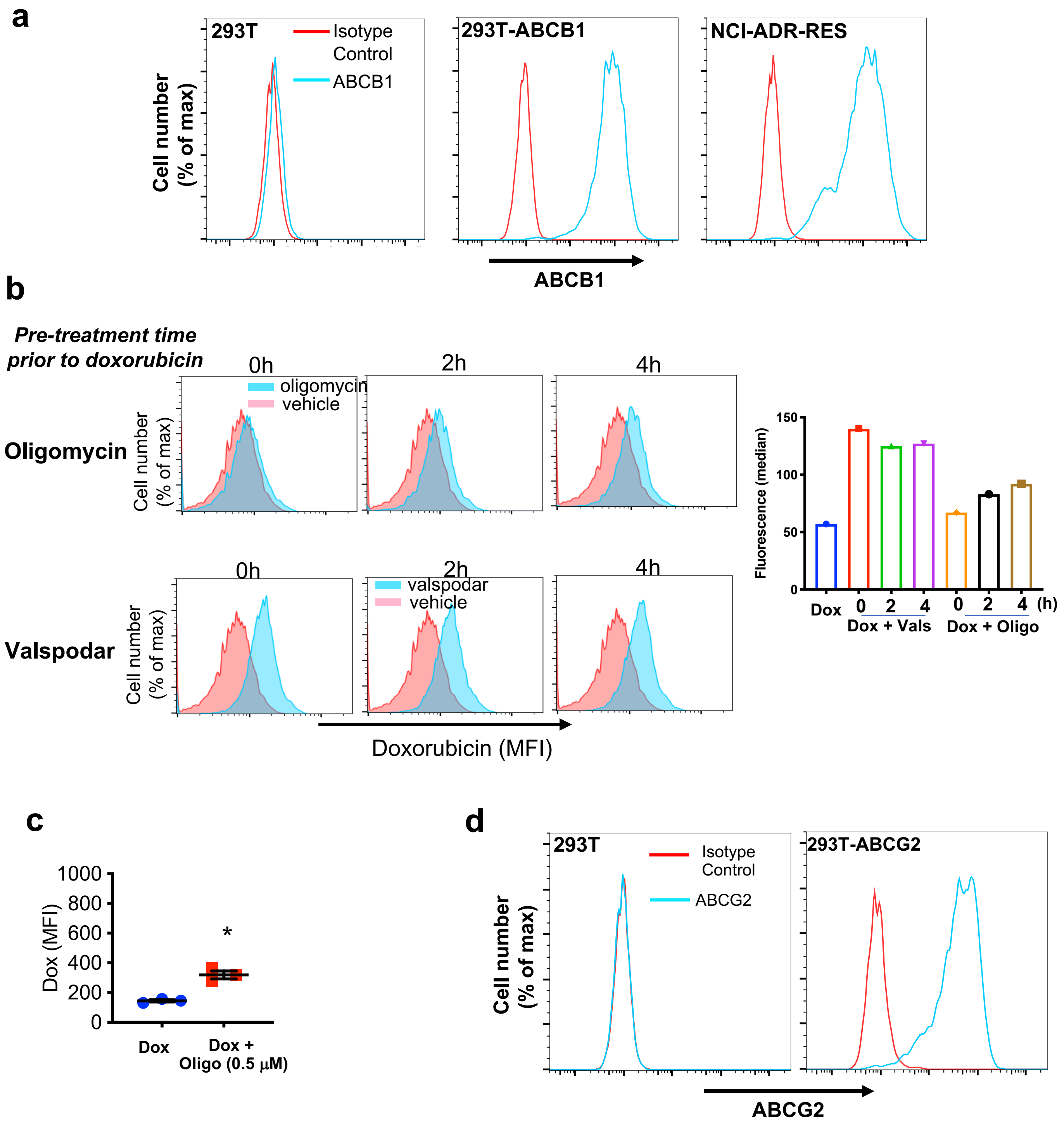

Supplementary Figure 9. Cell surface expression of ABC transporters. (a) HEK 293T control cells, ABCB1-expressing 293T cells and NCI/ADR-RES cells were stained with an anti-ABCB1 Ab (UIC2 clone) (blue) or an isotype control (red) and examined by flow cytometry analyses. **(b)** NCI/ADR-RES cells were incubated with oligomycin (5 μM, blue histograms upper panels) or Valspodar (1 μM, blue histograms lower panels) 2h or 4h prior to or simultaneously (0h) to doxorubicin (3 μM). Cells receiving only doxorubicin are shown as pink histograms. 2 h after doxorubicin administration cells were harvested and analyzed for doxorubicin fluorescence by flow cytometry. Right panel show the numerical value for the median of the histogram. (**c**) NCI/ADR-RES cells were treated with 0.5 μM of oligomycin for 3 h, and then incubated with doxorubicin for 2 h. Doxorubicin fluorescence was examined by flow cytometry. n=3, * denotes p=0.003 by unpaired *t* test. Mean +/- SEM is shown. (**d**) HEK 293T control cells and ABCG2-expressing 293T cells were stained with an anti-ABCG2 Ab (5D3 clone) (blue) or an isotype control (red) and examined by flow cytometry analyses.

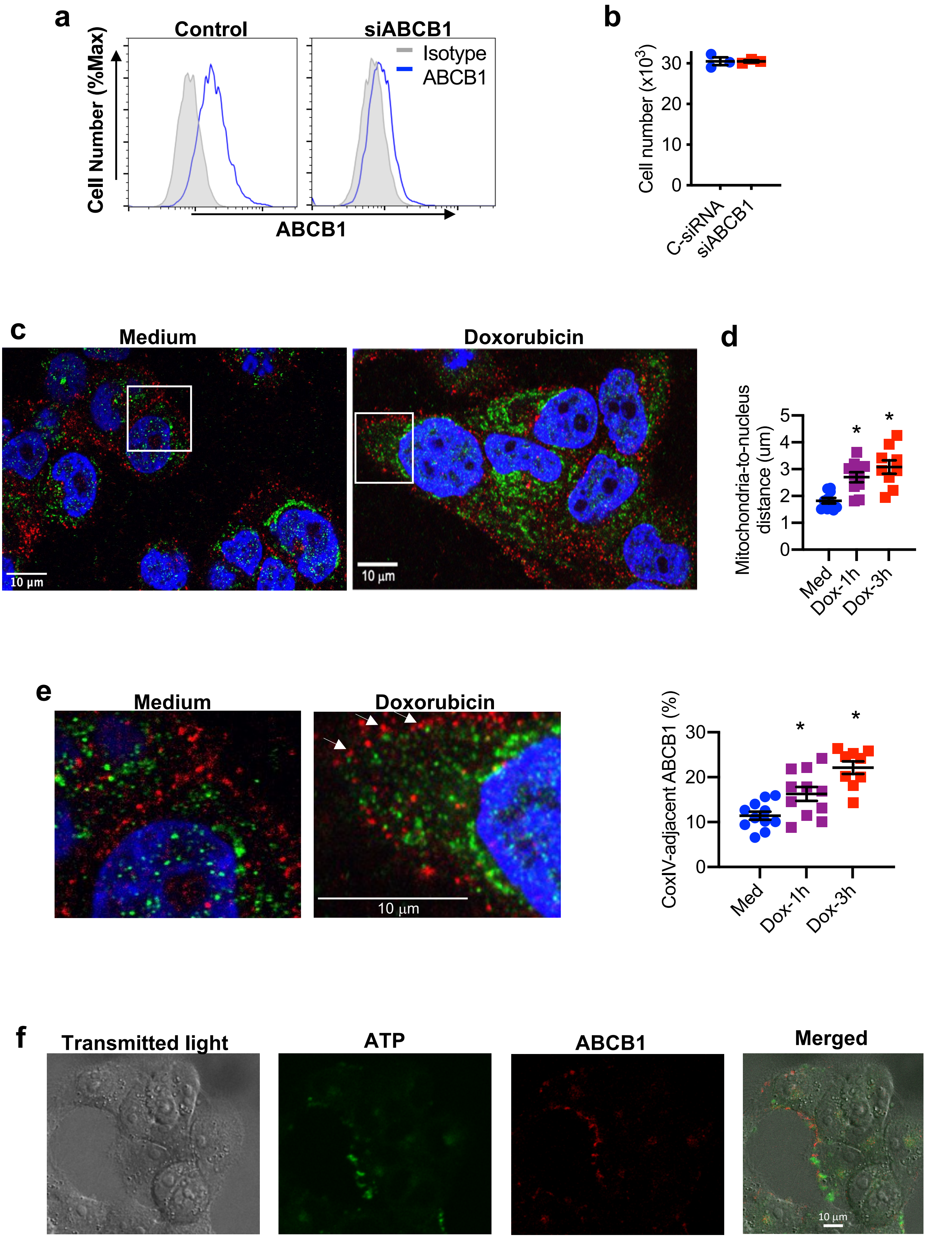

Supplementary Figure 10

g

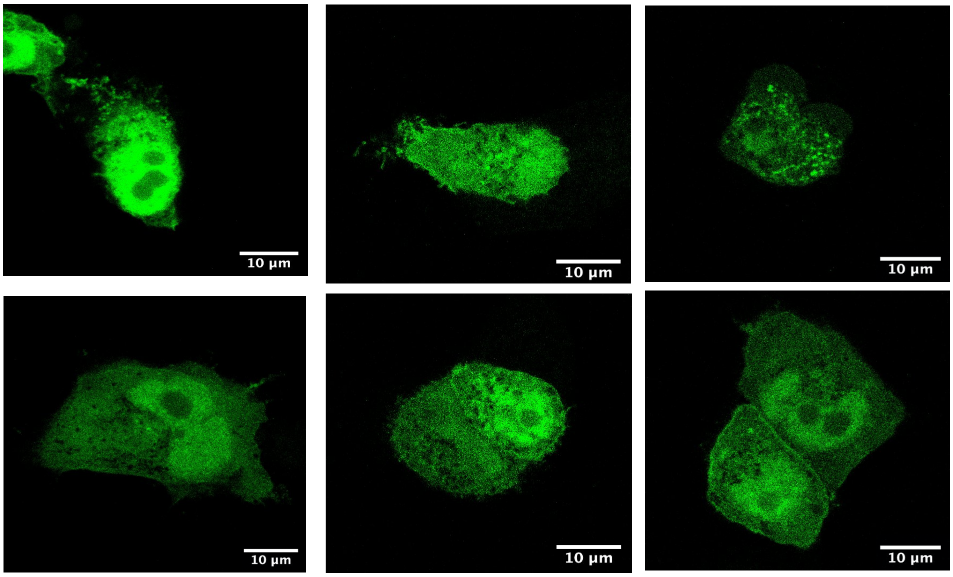

Supplementary Figure 10. Subcellular localization of mitochondria, ATP-rich microdomains and ABCB1 in NCI/ADR-RES cells. (**a**) NCI/ADR-RES cells were transfected with a siRNA for ABCB1 (siABCB1) or control and after 48 h cells were staining for ABCB1 (blue histograms) or with an isotype Ab control (black histograms) and were examined flow cytometry. **(b)** NCI/ADR-RES cells were transfected as in (a) with a siRNA for ABCB1 (siABCB1) (n=3) or control (n=3) and after 48 h cell number was determined by trypan blue staining. p=0.9873 by unpaired *t* test. **(c)** NCI/ADR-RES cells were treated with medium or doxorubicin (Dox) (1 μM) for 1 h or 3h, washed, fixed, permeabilized and stained for CoxIV as a marker for mitochondria (green) and ABCB1 (red), and examined by confocal microscopy (100x magnification). DAPI was used as nuclear marker (blue). Enlarged images of the insets in the lower panels are also shown. (**d**) Distance (μm) between mitochondria and nucleus in NCI/ADR-RES cells treated with medium (n=10) or doxorubicin (1 μM) for 1 h (n=10) or 3 h (n=9). Mean of the distance for each image (10 images for Med and Dox-1h and 9 images for Dox-3h, with 5-8 cells per image) is provided. p=0.0056, 0.0002 by one-way ANOVA and Tukey's multiple comparisons test. (**e**) Quantification (right panel) of the fraction (as %) of ABCB1 (red) on cell surface of the cells that was adjacent to a mitochondria as determined by CoxIV staining (green) in cells treated with Medium or doxorubicin for 1h or 3 h. Left panels display images showing a representation of ABCB1 molecules defined as being adjacent to CoxIV (white arrows). The images represent a magnification of the insets in the images shown in (c). Mean of the distance for each image (medium n=11, Dox-1h n=11 and Dox-3h n=9) with 5-8 cells per image is provided. p=0.0301, 0.0001 by one-way ANOVA and Tukey's multiple comparisons test. **(f)** Individual images for ATP probe (green), ABCB1 (red) and transmitted light of the merge image shown in Fig. 3k for NCI/ADR-RES cells. (**g**) NCI/ADR-RES cells were transfected with the iATPSnFR10 ATP reporter plasmid and after 36 h cells were treated with vehicle or oligomycin for 4 h. Cells were then fixed and visualized by confocal microscopy. Shown images are representative of two independent experiments. * denotes p< 0.5 by unpaired *t* test or one-way ANOVA and Tukey's multiple comparisons test. Mean +/- SEM is shown for all figures.

a

Amino acid sequences of MCJ mimetics (N to C)	
MITOx20	YGKKRRQRRGMAARGVIAPVGESLRYAEYLGTRTWVPKGLKSP
Control-20	YGKKRRQRRGMAARGVIAPVGESLRYAEYL
MITOx30	$R_DF_XR_DF_XR_DF_XR_D$MAARGVIAPVGESLRYAEYL
Control-30	$R_DF_XR_DF_XR_DF_XR_D$LYEAYRLSEGVPAIVGRAAM
MCJ N-terminus (first 20 aa), underlined; R_D, D-Arginine; F_X, Cyclohexylalanine	

b

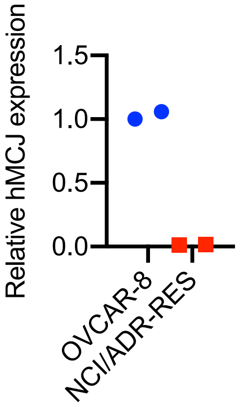

c

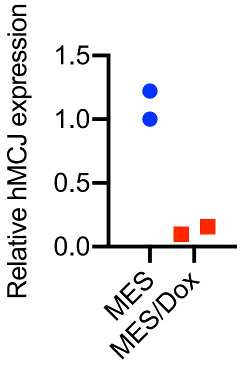

Supplementary Figure 11. (a) Amino acid sequences of N-MCJ mimetics. (**b**) Relative human MCJ (DnaJC15) mRNA levels in OVCAR-8 and NCI/ADR=RES cells by real time RT-PCR using HPRT as housekeeping gene. (**c**) Relative human MCJ expression in MES and MES/Dox cells by real time RT-PCR as in (b) n=2.

a

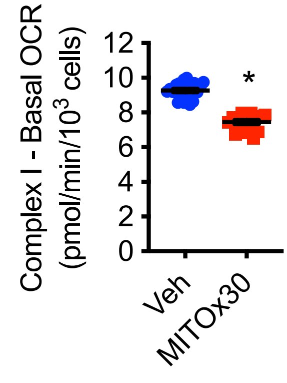

b

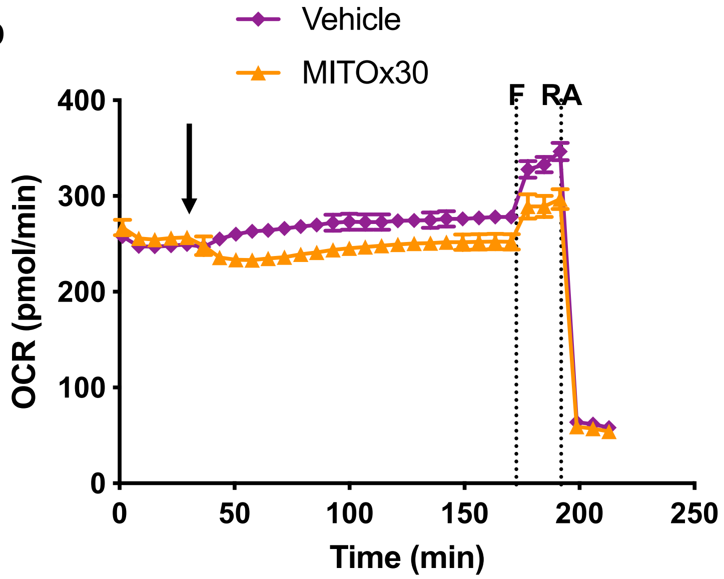

Supplementary Figure 12. (a) NCI/ADR-RES cells were treated with MITOx30 (25 mM) (n=18) or vehicle (n=21) for 12 h and Complex I activity was determined using the seahorse assay. * denotes p=0.0001 by unpaired *t* test. **b)** MCF7 cells were analyzed for OCR at baseline and in response to sequential injections of vehicle or MITOx30 (25 μM), FCCP (F), and rotenone with antimycin (RA) by Seahorse Mitostress assay (vehicle n=6, MITOx30 n=6). Mean +/- SEM is shown for all figures.

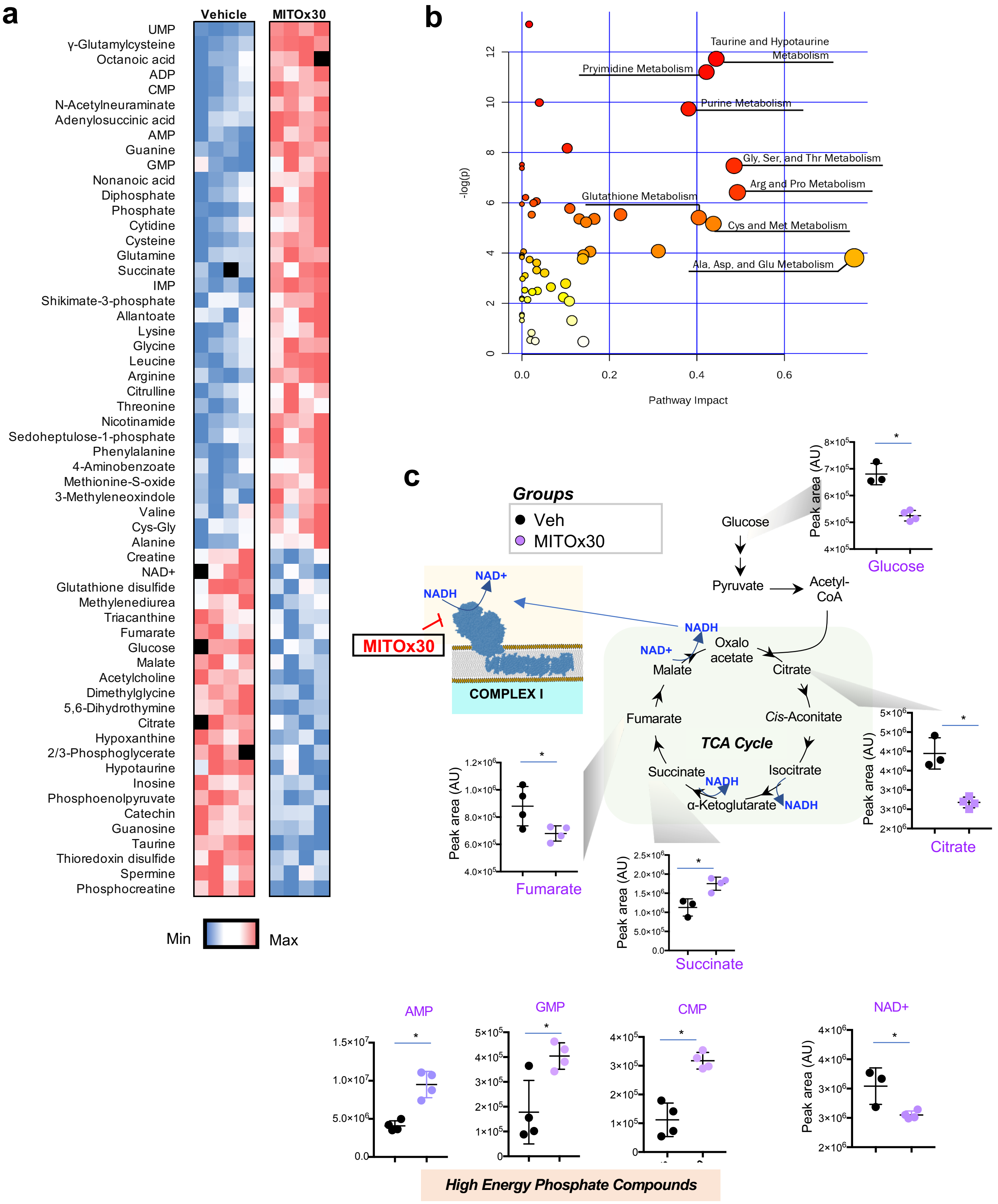

Supplementary Figure 13

Supplementary Figure 13. (a) NCI/ADR-RES cells were treated with MITOx30 (25 μM) for 12 h and then the relative abundances of metabolic intermediates compared to vehicle treated cells were determined by mass spectrometry based metabolomics. Within each cell type each column represents a independent cell preparation (n=4 for each cell type). Color represents the actual value with intense blue representing the lower value intense red the highest value. **(b)** Summary plot of Metabolite Sets Enrichment Analysis (MSEA). Altered metabolic pathways in NCI/ADR-RES cells after treatment with MITOx30, based on the statistically significant metabolites (t-test). The p values for the metabolic pathways are color coded, white representing the least significant, and red the highly significant. Pathway impact represents the number of metabolites that support the assignment of a pathway, with higher number of metabolites associated with higher pathway impact. (**c**) Peak area values (AU, arbitrary units) of some of the metabolites from the studies in (a) that are affected by MITOx30. n=4. for each group, Mean ± SD shown. * denotes $p< 0.05$ by unpaired one-sided *t* test.

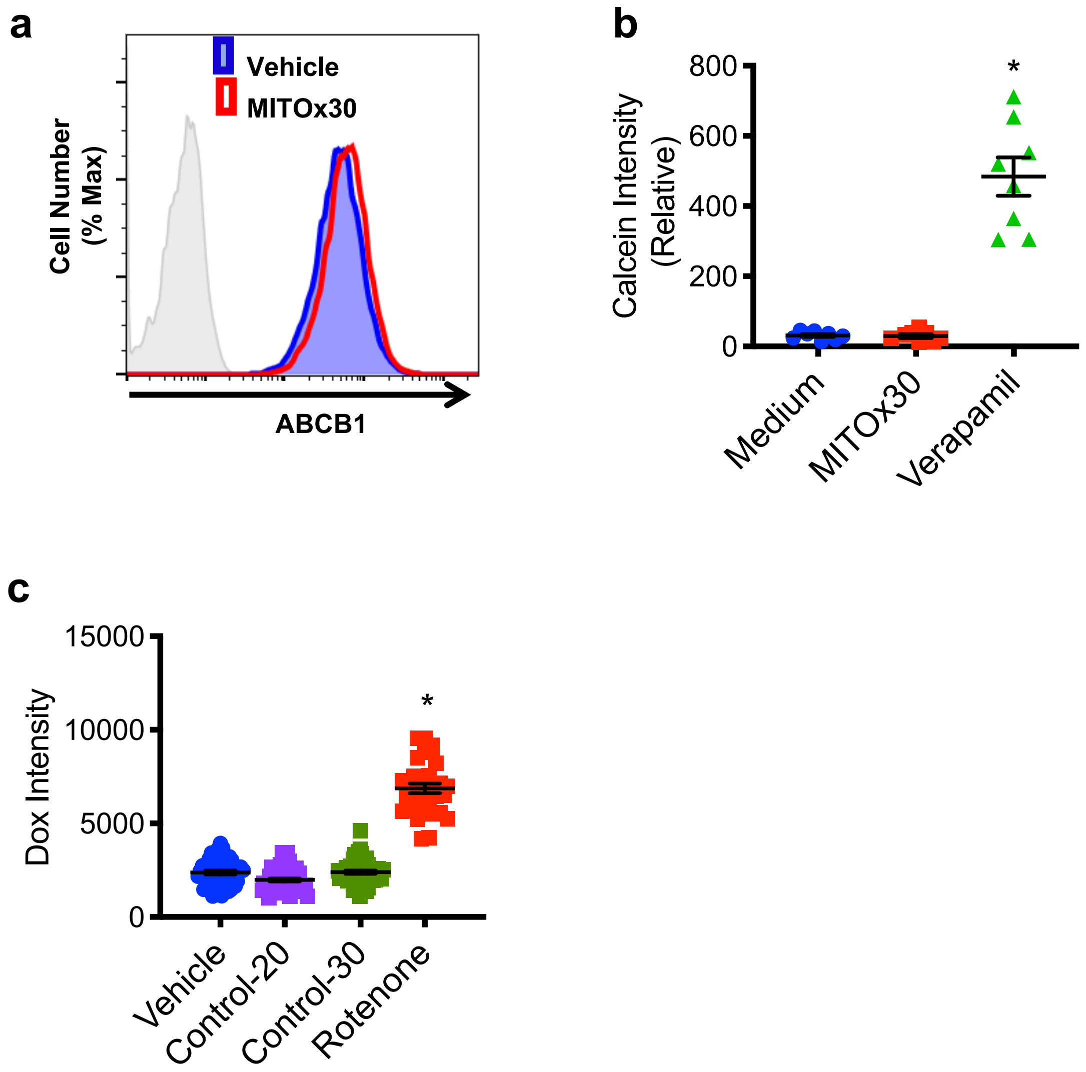

Supplementary Figure 14. (a) NCI/ADR-RES cells were treated with vehicle or MITOx30 (5µM) for 24 h. Cells were harvested, stained for ABCB1 and analyzed by flow cytometry. Isotype control staining is shown in gray histogram. (**b**) NCI/ADR-RES cells (n=8) were incubated with calcein in the presence of MITOx30 (20 µM) or verapamil (20 µM). Calcein fluorescence in the cells was determined after 20 min. p=0.9992, 0.0001 by one-way ANOVA and Tukey's multiple comparisons test. (**c**) NCI/ADR-RES cells were incubated with vehicle (n=51), Control-20 (5 µM) (n=51), Control-30 (5 µM) (n=51) or rotenone (50 µM) (n=32) for 2 h followed by incubation with doxorubicin (Dox, 3 µM) for 3 h. Cells were then fixed, stained for nuclear marker and analyzed by confocal microscopy as described in Fig. 6C. p= 0.0742, 0.9997, 0.0001 by one-way ANOVA and Tukey's multiple comparisons test. Mean +/- SEM is shown. * denotes p<0.05 as determined by one-way ANOVA and Tukey's multiple comparisons test.

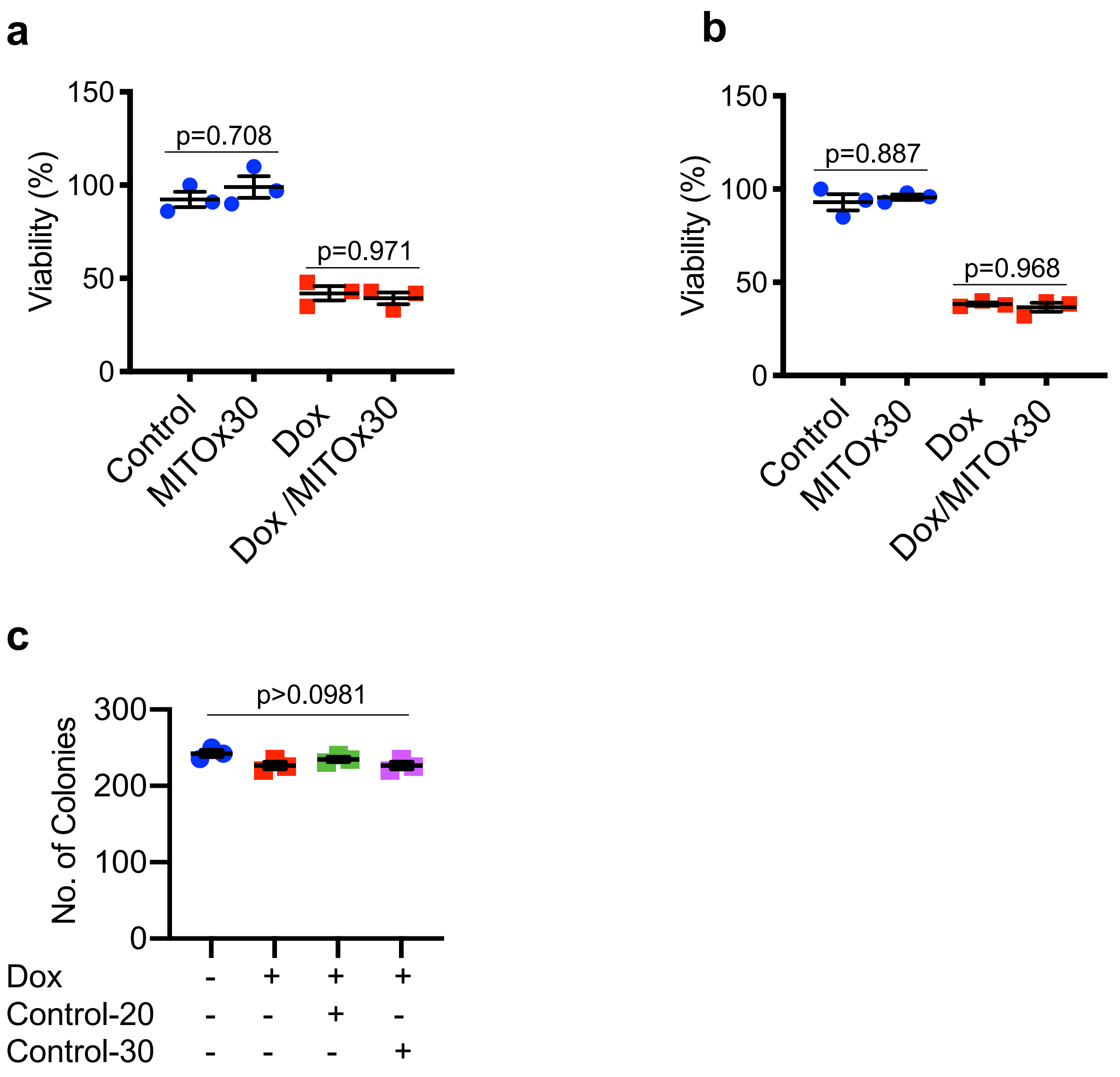

Supplementary Figure 15. (**a**) MCF7 cells (p=0.7079, 0.9709) and (**b**) OVCAR8 cells (p=0.8866, 0.9678) were treated with doxorubicin (3 μM), Control-30 (5 μM), and/or MITOx30 (5 μM) for 3 d and then cell viability was determined by Trypan blue staining (n=4). (**c**) NCI/ADR-RES cells were treated with doxorubicin (3 μM) in combination with Control-20 (5 μM) or Control-30 (5 μM) for 2 d, replated at a low density (400 cells), grown in normal culture medium for 1 wk, and then the number of colonies formed was determined (n=3). p= 0.0981, 0.5692, 0.0981. Mean +/- SEM is shown. p values determined by one-way ANOVA and Tukey's multiple comparisons test.

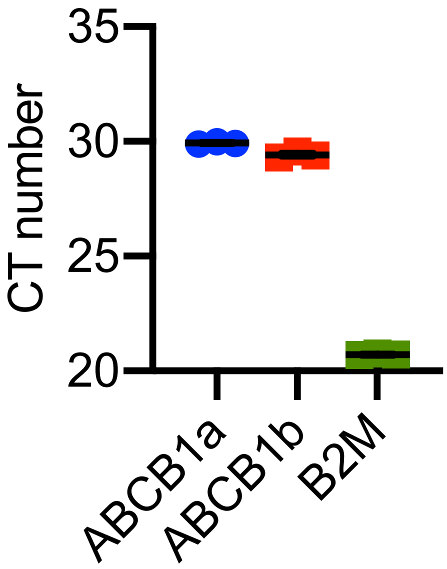

Supplementary Figure 16. ABCB1 expression in mouse mammary tumors. Enriched tumor cell preparation was obtained from mammary tumors of MCJ KO MMTV mice after digestion using the Tumor Dissociation kit (Miltenyi). ABCB1A and ABCB1B mRNA levels were determined by real time RT-PCR. β2microglobulin expression was used as positive control. Cycle Threshold (CT) number is shown (n=3). p=0.0004, 0.0001, 0.0001 by one-way ANOVA and Tukey's multiple comparisons test. Mean +/- SEM is shown.

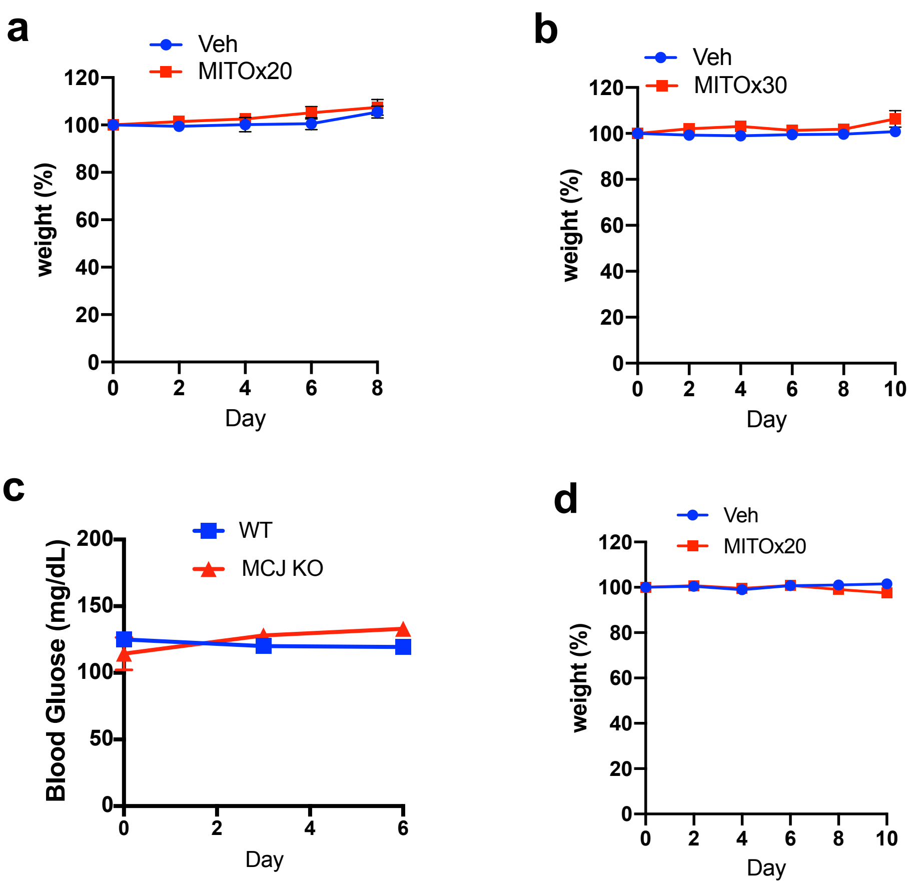

Supplementary Figure 17. (**a, b and d**) MITOx20 or MITOx30 (10 mg/Kg) were administered s.c. every other day to (**a**) MCJ KO mice (vehicle n=3, MITOx20 n=5), (**b**) wild type mice (vehicle n=5, MITOx30 n=3), or (**d**) NSG mice (vehicle n=1, MITOx20 n=5). Percentage weight relative to the weight before the treatment is provided. (**c**) WT (n=3) and MCJ KO mice (n=3) were treated with MITOx30 (10 mg/Kg) every other day. Blood glucose was determined prior the initiation of the treatment, 3 days and 6 days after the initiation of the treatment. Mean +/- SEM is shown.

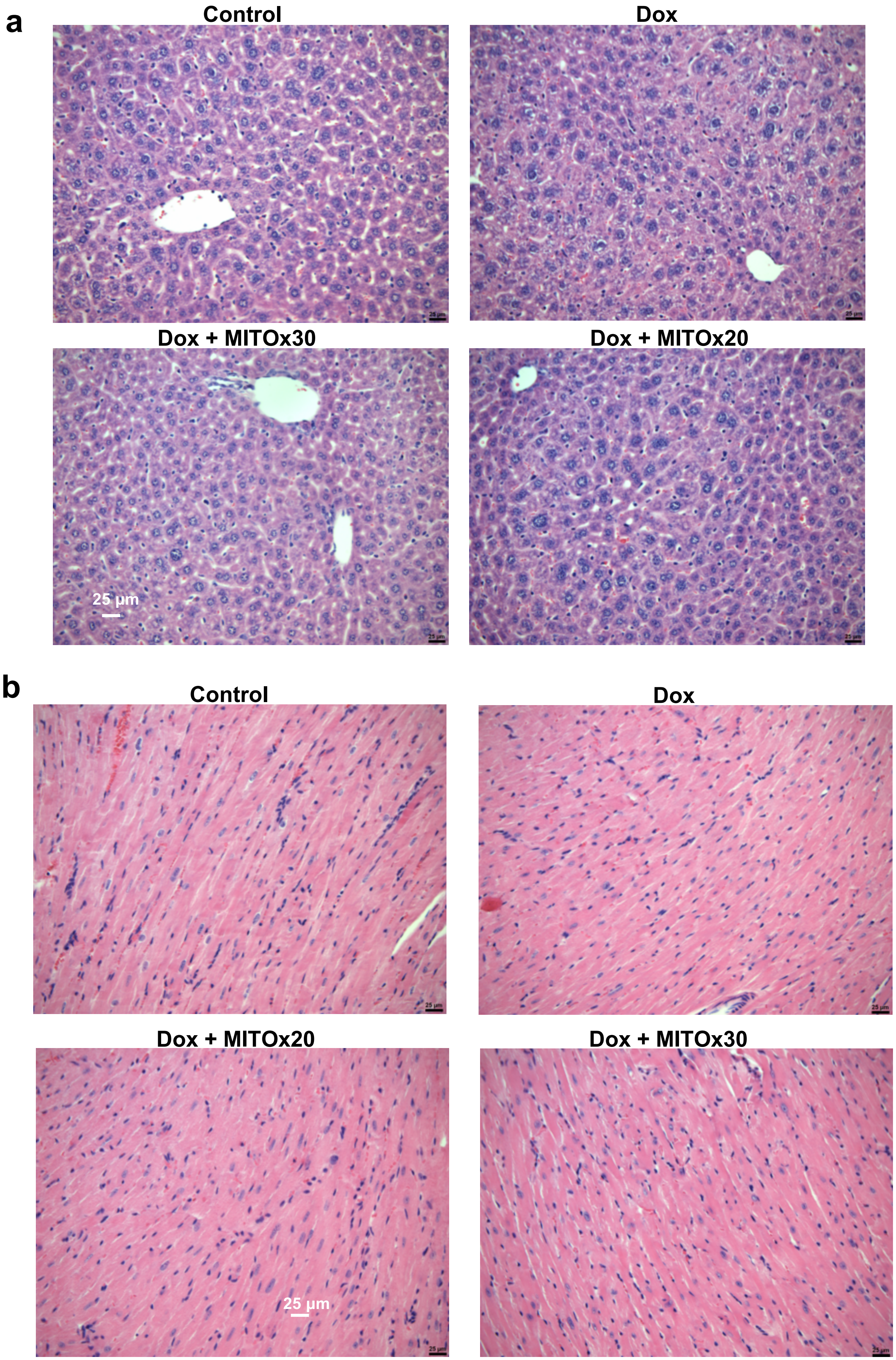

Supplementary Figure 18

Supplementary Figure 18. (a and b) Representative images (out of three mice per group) from hematoxylin and eosin (H&E) staining of (**a**) liver and (**b**) heart sections from control (untreated) mice or mice treated with doxorubicin (Dox) alone or in combination with MITOx20 or MITOx30. Treatments were performed every other day. Mice were sacrificed after 11 d.

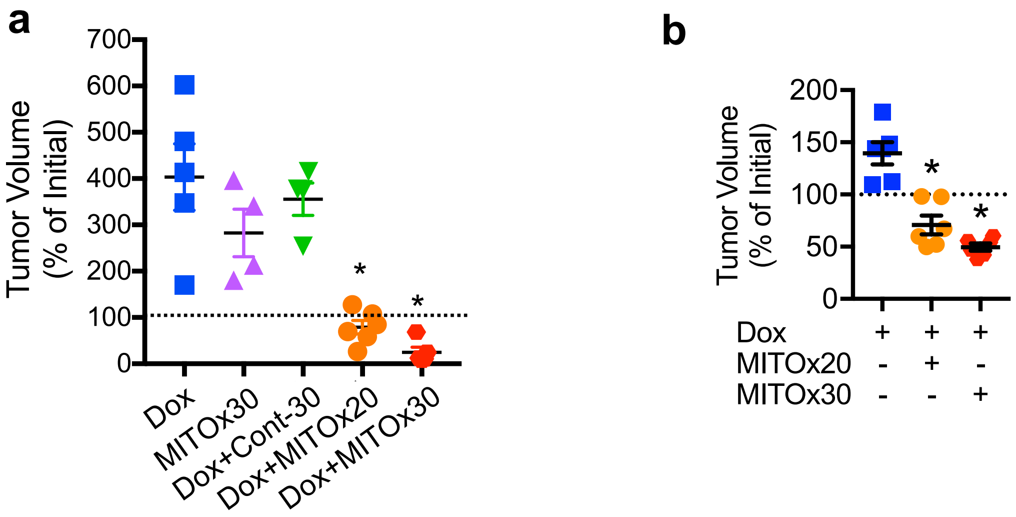

Supplementary Fig. 19. (**a**) MCJ-deficient MMTV-PyMT mice were treated with doxorubicin alone (Dox, n=5), MITOx30 alone (n=4), or doxorubicin in combination with Control-30 (n=4), MITOx20 (n=6), or MITOx30 (n=5) every other day for 12 d. Tumor volumes at the end of treatment relative to the initial size is shown. p=0.1445, 0.8567, 0.0001, 0.0001. (**b**) NSG mice with NCI/ADR-RES cell xenografts were treated with doxorubicin alone or in combination with MITOx20 or MITOx30 every other day for 8 d (n=6). Tumor volumes at the end of treatment relative to the initial size. Dotted lines represent initial tumor volumes prior to treatment (100 %). p=0.0001, 0.0001. Mean ± SEM provided. * denotes p< 0.05 by one-way ANOVA and Tukey's multiple comparisons test.
