# Supplementary material for: Mitochondrial ATP fuels ABC transporter-mediated drug efflux in cancer chemoresistance
Source: Nat Commun. 2021 May 14;12:2804. doi: 10.1038/s41467-021-23071-6 (PMC8121950; doi:10.1038/s41467-021-23071-6)
Supplement: Supplementary file 3 — Reporting summary [file 41467_2021_23071_MOESM3_ESM.pdf]

natureresearch

Corresponding author(s): Mercedes Rincon

Last updated by author(s): Mar 25, 2021

Reporting Summary

Nature Research wishes to improve the reproducibility of the work that we publish. This form provides structure for consistency and transparency in reporting. For further information on Nature Research policies, see Authors & Referees and the Editorial Policy Checklist .

Statistics

For all statistical analyses, confirm that the following items are present in the figure legend, table legend, main text, or Methods section.

n/a	Confirmed	
☐	☒	The exact sample size (*n*) for each experimental group/condition, given as a discrete number and unit of measurement
☐	☒	A statement on whether measurements were taken from distinct samples or whether the same sample was measured repeatedly
☐	☒	The statistical test(s) used AND whether they are one- or two-sided<br>*Only common tests should be described solely by name; describe more complex techniques in the Methods section.*
☐	☒	A description of all covariates tested
☐	☒	A description of any assumptions or corrections, such as tests of normality and adjustment for multiple comparisons
☐	☒	A full description of the statistical parameters including central tendency (e.g. means) or other basic estimates (e.g. regression coefficient) AND variation (e.g. standard deviation) or associated estimates of uncertainty (e.g. confidence intervals)
☐	☒	For null hypothesis testing, the test statistic (e.g. *F*, *t*, *r*) with confidence intervals, effect sizes, degrees of freedom and *P* value noted<br>*Give P values as exact values whenever suitable.*
☒	☐	For Bayesian analysis, information on the choice of priors and Markov chain Monte Carlo settings
☒	☐	For hierarchical and complex designs, identification of the appropriate level for tests and full reporting of outcomes
☐	☒	Estimates of effect sizes (e.g. Cohen's *d*, Pearson's *r*), indicating how they were calculated

Our web collection on statistics for biologists contains articles on many of the points above.

Software and code

Policy information about availability of computer code

Data collection	No software or code was used in data collection.
Data analysis	Graphpad Prism, version 7, Statistical Analysis Software (SAS) and GENE-E (Broad Institute) were used to analyze data.

For manuscripts utilizing custom algorithms or software that are central to the research but not yet described in published literature, software must be made available to editors/reviewers. We strongly encourage code deposition in a community repository (e.g. GitHub). See the Nature Research guidelines for submitting code & software for further information.

Data

Policy information about availability of data

All manuscripts must include a data availability statement. This statement should provide the following information, where applicable:

- Accession codes, unique identifiers, or web links for publicly available datasets
- A list of figures that have associated raw data
- A description of any restrictions on data availability

All raw data and statistical analyses for metabolomics are provided in the supplementary tables. Original .mzxml data were uploaded to Metabolome Xchange. Data were deposited through metabolomics Xchange and the accession code numbers are ST001730, ST001731, ST001732

Field-specific reporting

Please select the one below that is the best fit for your research. If you are not sure, read the appropriate sections before making your selection.

☒ Life sciences ☐ Behavioural & social sciences ☐ Ecological, evolutionary & environmental sciences

Life sciences study design

All studies must disclose on these points even when the disclosure is negative.

Sample size	Pilot and published studies were used to determine the sample size. The mean and variance between groups was sufficient to determine if differences between groups were significant.
Data exclusions	Within the Seahorse analyses some exclusions were performed due to technical difference due to instrument (for instance, the known edge effect in plates or wells that may not get injected properly).
Replication	Multiple independent experiments were conducted to verify the reproducibility of the data. All attempts at reproducibility were successful.
Randomization	Mice were randomly assigned treatment groups independent of the initial size of the tumor.
Blinding	As a consequence of our randomization process, each animal cage contained both control and treated mice in an unpredictable order.

Reporting for specific materials, systems and methods

We require information from authors about some types of materials, experimental systems and methods used in many studies. Here, indicate whether each material, system or method listed is relevant to your study. If you are not sure if a list item applies to your research, read the appropriate section before selecting a response.

Materials & experimental systems

n/a	Involved in the study	
☐	☒	Antibodies
☐	☒	Eukaryotic cell lines
☒	☐	Palaeontology
☐	☒	Animals and other organisms
☒	☐	Human research participants
☒	☐	Clinical data

Methods

n/a	Involved in the study	
☒	☐	ChIP-seq
☐	☒	Flow cytometry
☒	☐	MRI-based neuroimaging

Antibodies

Antibodies used	Anti-NDUFS3 (Abcam)<br>Anti-NDUFA9 (Abcam)<br>Anti-Actin (Santa Cruz)<br>Anti-ABCB1 (ThermoFisher, Biolegend)<br>Anti-ABCG2 (ThermoFisher)<br>Anti-human MCJ, generated by the Rincon group (Hatle et al, Mol Cell Biol 2007)<br>Anti-GAPDH (Santa Cruz)<br>Anti-AMPK and anti-phospho-AMPK (Cell Signaling)<br>Anti-Stat3 (BD BioScience)<br>Anti-CoxIV (Abcam)
Validation	The commercial anti-NDUFS3, NDUFA9, Actin, ABCB1,, ABCG2, CoxIV, AMPK, Stat3 antibodies gave a band of the expected molecular weight and were validated by the manufacturers. The anti-human MCJ was generated by the Rincon group (Hatle et al, Mol Cell Biol 2007) and validated for western blots using human cells that either expressed MCJ or had reduced MCJ expression because of siRNA.

Eukaryotic cell lines

Policy information about cell lines

Cell line source(s)	MCF7 - ATCC<br>NCI/ADR-RES - NCI<br>OVCAR8 - Dr. Ernst Lengyel at the University of Chicago<br>MES - Dr. Branimir Sikic at Stanford University<br>MES/Dox - Dr. Branimir Sikic at Stanford University<br>MCF7/Tx400 - Laboratory of Cell Biology at NCI<br>293T - Laboratory of Cell Biology at NCI

Authentication	MCF7 - Authenticated via GenePrint 10 Cell IDTM by Vermont Cancer Center DNA Facility<br>NCI/ADR-RES - Authenticated via GenePrint 10 Cell IDTM by Vermont Cancer Center DNA Facility<br>OVCAR8 - Authenticated via GenePrint 10 Cell IDTM by Vermont Cancer Center DNA Facility<br>MES - Not authenticated<br>MES/Dox - Not authenticated<br>MCF7/Tx400 - Authenticated at NCI<br>293T - Authenticated at NCI
Mycoplasma contamination	Cells tested negative for mycoplasma contamination.
Commonly misidentified lines<br>(See ICLAC register)	NCI/ADR-RES cells is the currently accepted name from previously named MCF7/ADR cells

Animals and other organisms

Policy information about studies involving animals; ARRIVE guidelines recommended for reporting animal research

Laboratory animals	Female mice were 3-7 months of age. WT and MCJ deficient MMTV-PyMT (FBV background) and NSG strains
Wild animals	The study did not involve wild animals.
Field-collected samples	The study did not involve field-collected samples.
Ethics oversight	All mice were maintained in the animal facility at the University of Vermont and University of Colorado under Institutional Animal Care and Use Committee (IACUC) approved conditions.

Note that full information on the approval of the study protocol must also be provided in the manuscript.

Flow Cytometry

Plots

Confirm that:

- [x] The axis labels state the marker and fluorochrome used (e.g. CD4-FITC).
- [x] The axis scales are clearly visible. Include numbers along axes only for bottom left plot of group (a 'group' is an analysis of identical markers).
- [x] All plots are contour plots with outliers or pseudocolor plots.
- [x] A numerical value for number of cells or percentage (with statistics) is provided.

Methodology

Sample preparation	Cells were pretreated with metabolic inhibitors as indicated for 2 h followed by the addition of doxorubicin (3 µM) or Hoechst 33342 (0.5 ug/mL) for 2 h. Cells were then washed, fixed in PBS supplemented with 1 % paraformaldehyde, and then immediately analyzed by flow cytometry analysis.
Instrument	BD Biosciences LSRII Flow Cytometer and Cytek Northern Lights Flow Cytometer
Software	FlowJo Software
Cell population abundance	No minority population occurred in this study. These are cell lines with a single population.
Gating strategy	Cell size by FSC/SSC gate followed by histograms for the single specific fluorochrome.

- [ ] Tick this box to confirm that a figure exemplifying the gating strategy is provided in the Supplementary Information.
